# Draft Genome Sequence of a Community-Associated Methicillin-Resistant Panton-Valentine Leukocidin-Positive *Staphylococcus aureus* Sequence Type 30 Isolate from a Pediatric Patient with a Lung Infection in Brazil

**DOI:** 10.1128/genomeA.00907-15

**Published:** 2015-08-20

**Authors:** Craig Stephens, Paul Jang-Yeon Cho, Veronica Afonso de Araujo, Ivete Martins Gomes, Selma Maria de Azevedo Sias, Claudete A. Araújo Cardoso, Lee W. Riley, Fábio Aguiar-Alves

**Affiliations:** a Biology Department and Public Health Program, Santa Clara University, Santa Clara, California, USA; b School of Public Health, University of California, Berkeley, Berkeley, California, USA; c Faculdade de Medicina, Programa de Pós Graduação em Ciências Médicas, Fluminense Federal University, Niterói, Rio de Janeiro, Brazil; d Departamento Materno-Infantil, Faculdade de Medicina, Fluminense Federal University, Niterói, Rio de Janeiro, Brazil; e Faculdade de Medicina, Programa de Pós Graduação em Patologia, Fluminense Federal University, Niterói, Rio de Janeiro, Brazil; f Laboratório de Epidemiologia Molecular e Biotecnologia (LEMB), Laboratório Universitário Rodolfo Albino, Fluminense Federal University, Niterói, Rio de Janeiro, Brazil

## Abstract

The sequence of methicillin-resistant *Staphylococcus aureus* strain B6 (sequence type 30 [ST30], *spa* type t433, staphylococcal chromosomal cassette *mec* element [SCC*mec*] type IVc, Panton-Valentine leukocidin [PVL] positive), isolated from a pediatric patient with a lung infection in Niterói, Rio de Janeiro, Brazil, is described here. The draft genome sequence includes a 2.8-Mb chromosome, accompanied by a 20-kb plasmid containing *blaZ* and two small cryptic plasmids.

## GENOME ANNOUNCEMENT

The emergence of community-associated methicillin-resistant *Staphylococcus aureus* (CA-MRSA) has contributed significantly to an increase in the global staphylococcal disease burden (1, 2). We present here the genome sequence of an unusual CA-MRSA isolate from Niterói, Rio de Janeiro, Brazil. The source of the isolate was a 17-month-old boy diagnosed with pneumonia and pleural effusion of one lung. The *S. aureus* strain (designated B6) isolated from drainage fluid was resistant to methicillin and cefepime. The strain was characterized by multilocus sequence typing as sequence type 30 (ST30), by *spa* typing as t433, and as staphylococcal chromosomal cassette *mec* element (SCC*mec*) type IVc by PCR. The boy’s HIV-positive aunt, living in the same household, was asymptomatically colonized by a strain typed identically to B6 that may have been the source of the boy’s infection. ST30 strains are a major source of high-risk staphylococcal infection globally (3) but are rare in Brazil, where the ST239 Brazilian epidemic clone (SCC*mec* type III) is epidemiologically dominant (4). ST30 strains carrying Panton-Valentine leukocidin (PVL), as B6 does, are often associated with necrotizing pneumonia, which causes high morbidity and mortality rates in children (5, 6). Strain B6 is also unusual as a *spa* type t433 strain; most Brazilian ST30 isolates are t318, suggesting that this strain had a distinct origin. The genome sequence was therefore determined for use as a reference in the future.

DNA preparation for whole-genome sequencing was done with the Qiagen DNeasy kit (Qiagen, USA). Preparation of Illumina-compatible libraries (with index tags) for 300-bp paired-end reads were conducted according to a standard protocol (Wafergen Biosystems). Libraries were sequenced on a MiSeq instrument using V3 chemistry. Read trimming and contig assembly and analysis were performed using Geneious software (Biomatters Ltd.). Annotation of the draft genome employed RAST (7). Plasmids, resistance genes, and virulence factors were initially identified using Web-based tools from the Center for Genomic Epidemiology (http://www.genomicepidemiology.org), and prophages were identified using PHAST (8).

The *S. aureus* B6 draft genome sequence was assembled into 71 contigs >1 kb in length, with a mean contig length of 39,889 bp, maximum contig length of 222,661 bp, and an *N*_50_ of 72,253 bp. The mean read coverage of the assembled contigs was approximately 25-fold. The contigs comprise a 2,808,089-bp chromosome, a 20,335-bp circular plasmid, and two small circular plasmids of 1,990 and 1,503 bp. The SCC*mec* type IVc element containing *mecA* was 21,398 bp in length. The genome contained two prophages associated with virulence factors ϕPVL (37 kb), containing the PVL subunit genes *lukF* and *lukS*, and a prophage highly similar to ϕNM-3 (44 kb), which contains the enterotoxin A (*sea*) and staphylokinase genes, as well as other known virulence factors (9). The 20-kb plasmid, which closely resembled pMW2 from CA-MRSA strain MW2 (10), contained *blaZ* (β-lactamase) and genes for heavy metal resistance. The B6 genome encodes numerous additional virulence factors potentially relevant to pulmonary infection, including the *ica* operon (for the synthesis of extracellular poly-*N*-acetyl-glucosamine for biofilm formation); adhesins binding elastin (*ebpS*), fibronectin (*fnbA*), and collagen (*cna*); and multiple staphylococcal enterotoxins, exotoxins, and superantigens (11).

### Nucleotide sequence accession numbers.

This whole-genome shotgun project has been deposited at DDBJ/EMBL/GenBank under the accession no. LDIT00000000. The version described in this paper is version LDIT01000000.
